# Patterns of microsatellite distribution across eukaryotic genomes

**DOI:** 10.1186/s12864-019-5516-5

**Published:** 2019-02-22

**Authors:** Surabhi Srivastava, Akshay Kumar Avvaru, Divya Tej Sowpati, Rakesh K. Mishra

**Affiliations:** CSIR - Centre for Cellular and Molecular Biology, Uppal Road, Hyderabad, 500007 India

**Keywords:** Microsatellites, Phylogenomics, SSR, Length preference, Tandem repeats, Evolution

## Abstract

**Background:**

Microsatellites, or Simple Sequence Repeats (SSRs), are short tandem repeats of 1–6 nt motifs present in all genomes. Emerging evidence points to their role in cellular processes and gene regulation. Despite the huge resource of genomic information currently available, SSRs have been studied in a limited context and compared across relatively few species.

**Results:**

We have identified ~ 685 million eukaryotic microsatellites and analyzed their genomic trends across 15 taxonomic subgroups from protists to mammals. The distribution of SSRs reveals taxon-specific variations in their exonic, intronic and intergenic densities. Our analysis reveals the differences among non-related species and novel patterns uniquely demarcating closely related species. We document several repeats common across subgroups as well as rare SSRs that are excluded almost throughout evolution. We further identify species-specific signatures in pathogens like *Leishmania* as well as in cereal crops, *Drosophila*, birds and primates. We also find that distinct SSRs preferentially exist as long repeating units in different subgroups; most unicellular organisms show no length preference for any SSR class, while many SSR motifs accumulate as long repeats in complex organisms, especially in mammals.

**Conclusions:**

We present a comprehensive analysis of SSRs across taxa at an unprecedented scale. Our analysis indicates that the SSR composition of organisms with heterogeneous cell types is highly constrained, while simpler organisms such as protists, green algae and fungi show greater diversity in motif abundance, density and GC content. The microsatellite dataset generated in this work provides a large number of candidates for functional analysis and for studying their roles across the evolutionary landscape.

**Electronic supplementary material:**

The online version of this article (10.1186/s12864-019-5516-5) contains supplementary material, which is available to authorized users.

## Background

Repetitive DNA in eukaryotic genomes can be broadly classified into interspersed and tandem repeats. Microsatellites, also known as Simple Sequence Repeats or SSRs, are short tandem repeats of 1–6 nucleotide DNA motifs. They comprise a significant portion of the genome in complex organisms, often surpassing the proportion of coding sequences [1]. SSRs contribute to 3% of the human genome [2], and display a non-random distribution in many genomes [1, 3]. They have high mutation rates due to polymerase slippage, with a bias towards elongation [4]. Due to their highly polymorphic nature, microsatellites have long been used as molecular markers in a variety of fields including genotyping [5], marker-assisted selection [6], linkage analysis [7], and forensics [8]. Though a majority of SSRs in genomes are present at intergenic and non-coding regions, a small proportion of SSRs occur within exons [3, 9]. Abnormal expansion of SSRs within exons is associated with several diseases in humans such as Huntington’s disease and Spinocerebellar Ataxia (reviewed in [10]).

Recent studies have focused on the role of SSRs in cellular processes such as the epigenetic regulation of gene expression [11–13] and genome organization [14]. Microsatellites are believed to be under selection pressure in genomes, apparent in their distribution and abundance which is much higher than expected by chance or random accumulation [4]. A comprehensive analysis of these elements across the evolutionary landscape can help identify functionally relevant SSRs but in silico studies have mostly been limited by the efficiency, exhaustiveness and sensitivity of the various SSR identification programs they utilize and can be compromised by the quality of the SSR datasets generated [15, 16]. A few studies [1, 3] have chosen a small subset of representative species across evolution to analyze their SSR content but the results may reflect trends that are specific to the chosen species rather than the group they represent, particularly if the sequence quality of the available genomes is variable. Other studies [17–20] have limited their analysis to a single taxonomic group, making their observations difficult to understand in terms of the broader evolutionary landscape. These issues can be overcome using an unbiased examination of the genome-wide distribution patterns of microsatellites in related clades, with large-scale comparisons revealing potentially relevant trends.

Here, we have identified microsatellites from 719 eukaryotic species and studied their abundance across 15 taxonomic subgroups from protists to mammals, spanning 1.7 billion years of evolution. Our analysis reveals a large number of novel taxon-specific SSR signatures, and evolutionary differences in the context of SSR length, GC content and size of the repeat motifs. Interestingly, the microsatellite trends accurately reflect phylogeny and we posit that they can be useful in understanding evolutionary relationships. Finally, we have used the available genome annotation data from 334 species to understand the distribution of SSRs in coding and non-coding regions and report several novel observations that open up avenues for further experimental scrutiny.

## Results

### Overview of SSR distribution

**Fig. 1:**
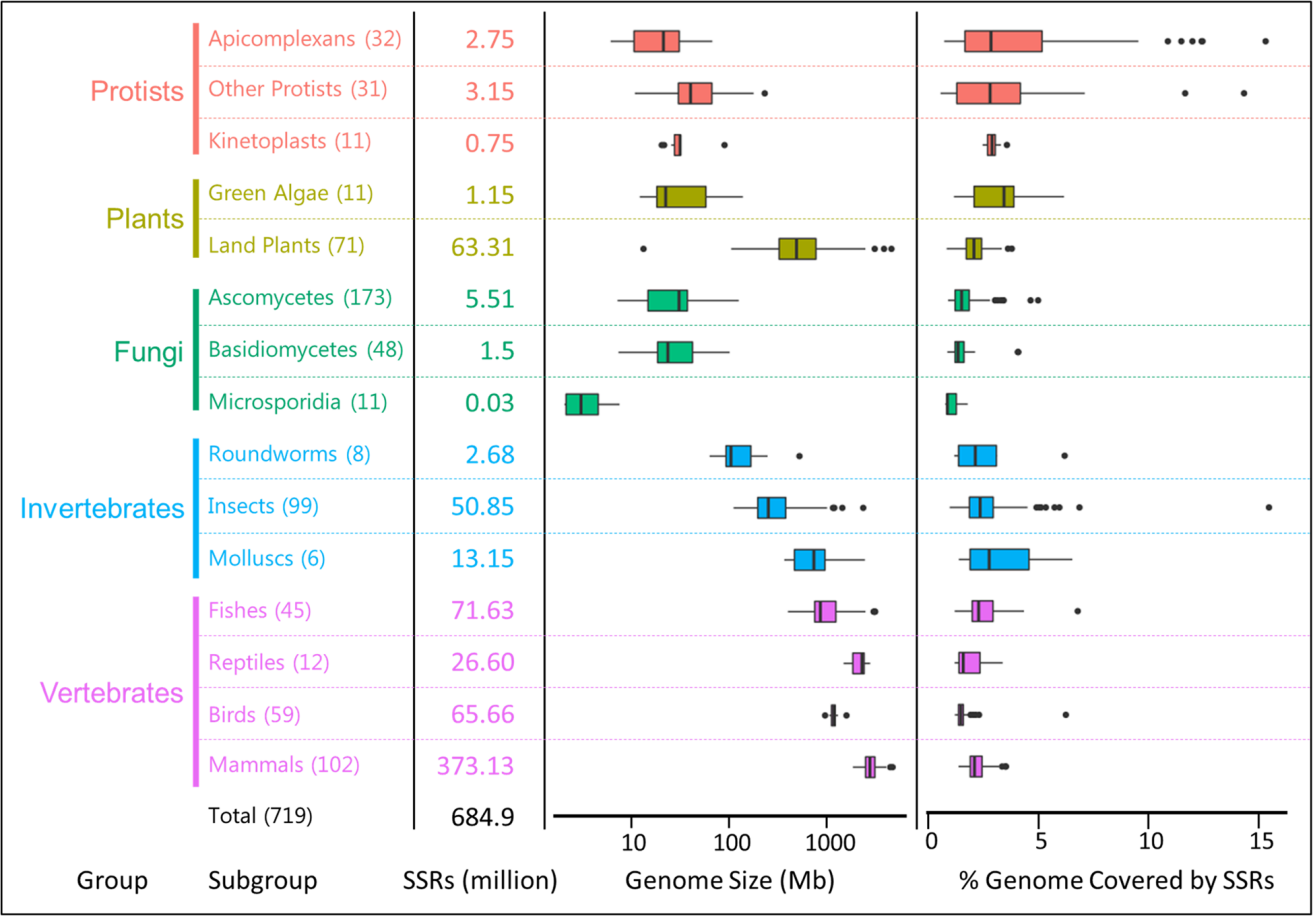
Overview of SSRs analyzed in this study. Approximately 685 million perfect SSRs (at least 12 bp in length) were identified from 719 eukaryotes across 15 subgroups, color coded and divided into 5 groups. Numbers in parenthesis indicate the number of organisms in each subgroup. The total number of SSRs (in millions) analyzed in each subgroup is indicated. Box plots indicate the distribution of genomic sizes (highly variable across taxonomic groups, left) and the SSR coverage (% of genome covered by SSRs which is relatively uniform, right) in each subgroup

We utilized our exhaustive repeat finding algorithm, PERF [15] to search for all 501 possible SSR motifs occurring in 719 eukaryotic organisms for which genome sequence is available in the NCBI database (see Methods). We identified a total of 684,885,656 perfect SSRs (length > = 12 bp) and analyzed their distribution patterns across organisms divided into 5 main groups (protists, plants, fungi, protostomes and deuterostomes) constituting 15 subgroups (Additional file 1: Table S1). Figure 1 summarizes the distribution of SSRs across each taxonomic group and their genomic relationships. As expected, the total SSR abundance is correlated with genome size (Additional file 2: Figure S1A, Pearson, r = 0.96). The top 50 organisms with high SSR frequency are mostly mammals including humans (4.6 million SSRs), a plant (*Aegilops tauschii*) and some fish (salmon species and coelacanth), each containing 4–5 million SSRs and with genome sizes > 2.2 Gb. Of note, the highest number of SSRs are found in the octopus (7.6 million; genome size 2.3 Gb), the only non-vertebrate animal in the top 50 of the SSR abundance table (Additional file 1: Table S1). At 1181 SSRs, the fungus *Encephalitozoon romaleae* (subgroup microsporidia) has the lowest number of SSRs, which correlates well with the fact that this pathogen has one of the smallest genomes studied (2.2 Mb). In order to normalize their occurrence to the genome size we looked at the density of SSRs (i.e. bp covered by SSRs per Mb of the genome). We found that unlike SSR abundance, there is no correlation between the SSR density and the genome size (Additional file 2: Figure S1A; Pearson, r = − 0.04), though land plants do show a slight negative correlation (Pearson, r = − 0.43) as documented previously [19].

**Table 1:** Uniquely abundant SSRs showing species-specific enrichment

S.No	Species	Uniquely abundant SSRs	Estimated Divergence Time from Common Ancestor
1	*Leishmania* species	AGGG^**^, AGGGG^**^, AGGGGG^**^, ACACGC^**^	1660 Ma
2	Green algae	CG^*^, ACGCG^*^, CCCCG^*^, ACGGCG^*^, ACGCCG^*^, AGCGCG^*^, ACGCGG^*^, ACGTCG^*^	1160 Ma
3	Cereals	CCGGCG^*^, CCCGCG^*^, ACGGCC^*^	104 Ma
4	*Drosophila* species	AACAGC^***^	127 Ma
5	Birds	AAACC^***^, AAAGG^***^, AAAACC^***^, AAAAGG^***^	111 Ma
6	Ruminants	AACTG^***^, AAAGTG^***^, AAGCTG^***^	56 Ma
7	Primates	AATGG^**^, ACCTCC^***^	67 Ma

At a density of 155 Kb/Mb the human body louse (*Pediculus humanus corporis*) is the top ranked organism in terms of SSR abundance per Mb of the genome, i.e. 15.5% of its genome is covered by SSRs. A recent analysis also made a similar observation, albiet from a comparison of only insect genomes [20]. At 21 Kb/Mb (SSR coverage 2.13%), humans have almost an order of magnitude lower SSR density than their parasitic louse. Among mammals, the kangaroo rat (*Dipodomys ordii*) has the highest density (34 Kb/Mb) followed closely by the house mouse (*Mus musculus*; 33.7 Kb/Mb). As a subgroup, mammals show little variance in SSR densities (about 3 fold difference between the highest and lowest SSR densities within mammals) (Additional file 1: Table S1, Sheet 2).

Protists, on the other hand, occupy the highest density ranges and show the greatest variance among individual organismal SSR densities - upto 27 fold difference between the highest and lowest SSR densities among all protists (*p* < 2.2e-16, F Test, Additional file 2: Figure S1B). For example, the *Dictyostelium* species (SSR density 143 Kb/Mb, SSR coverage 14.3%) and the *Plasmodium* species of the Apicomplexans subgroup (SSR density 124 Kb/Mb) have the highest SSR densities among all groups examined while those belonging to the Oomycetes class and the *Entamoeba* species have significantly lower SSR densities such as *Giardia lamblia* (SSR density 5228 bp/Mb) and *Aphanomyces invadans* (SSR density 4070 bp/Mb, only 0.4% of the genome covered by SSRs); in fact they occupy the lowest end of the density spectrum among all 719 species (Additional file 1: Table S1). The *Eimeria* species of protists from the Apicomplexans subgroup also have a high SSR density (average density 118 Kb/Mb) and these SSRs are notably GC-rich (SSR GC range of around 60%). It is interesting to note that these protists have such a high SSR density despite their small genome size. Fungi have the lowest average SSR density among all the subgroups while among vertebrates, birds have the lowest average, again with very little variance (Additional file 1: Table S1, Sheet 2), with the exception of the collared flycatcher (*Ficedula albicollis*) that has a very high SSR density of 61 Kb/Mb (SSR frequency of 1.27 million, 6.1% coverage in a genome size of 1.1 Gb). Further analysis of SSR content in this genome revealed multiple instances of long C/G stretches (upto 4 kb) which are likely to be assembly errors creating an artifact in the SSR density calculations.

The GC% of the SSRs is well correlated with the genomic GC content (Additional file 2: Figure S2A, Pearson, r = 0.94) and shows some interesting subgroup-specific patterns (Additional file 2: Figure S2B). Green algae (olive green arrow) and a few protists (*Aureococcus anophagefferens, Emiliania huxleyi, Thecamonas trahens*) with GC-rich genomes (genomic GC 55–65%) have an abundance of GC-rich SSRs (SSR GC range 75–100%). SSRs of intermediate GC content (SSR GC range 50%) are abundant in fungi. Protostomes and deuterostomes, however, have uniformly AT-rich SSRs (SSR GC range < 25%). We note that there is no correlation between the overall SSR abundance of an organism and its genomic GC content (Additional file 2: Figure S1A).

### SSR abundance trends across evolution

**Fig. 2:**
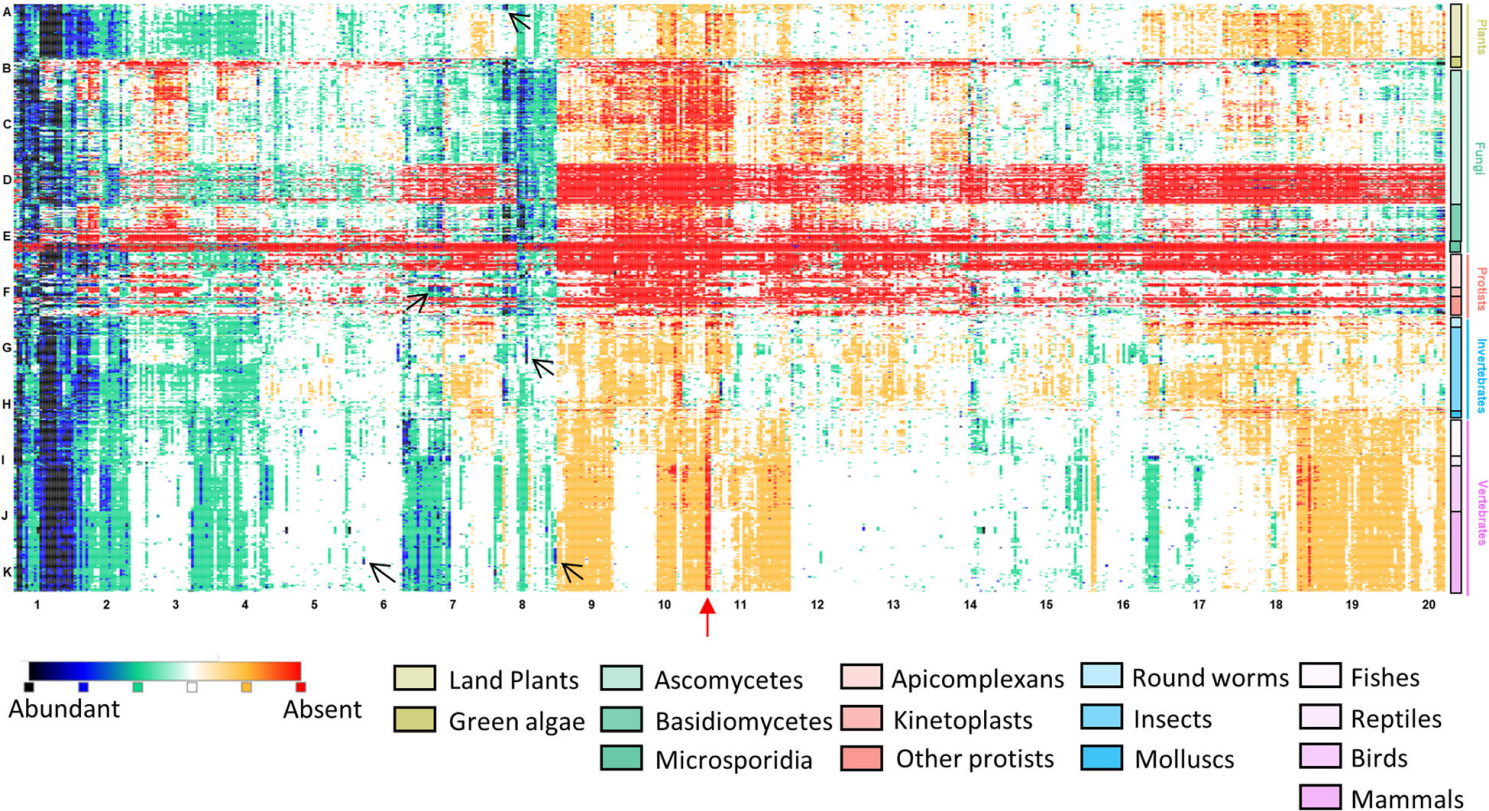
Enrichment trend of the 501 SSRs across 719 genomes. SSRs in an organism are ranked based on their density, defined as the total number of bp covered by the SSR in an organism normalized to genome size (that is, bp covered/Mb). The density based ranking is used to generate a heat map as per the color scale indicated. The 501 SSRs are arranged in columns and the 719 organisms are arranged in rows (the 5 main groups are indicated on the right; subgroups are indicated below the heatmap). Alphabets on the left and numbers at the bottom of the heatmap indicate approximate positions of the tiles in a virtual grid (eg., A1 to K20). Arrows mark the positions for uniquely abundant (enriched) SSR signatures described in the text and in Table 1

We plotted a ranked SSR density heat map (Fig. 2, see methods) to look at density-based abundance trends of the 501 SSRs (columns) across all the 719 genomes (rows). SSRs were considered abundant in an organism if they occurred in the top 10 (black tiles) or top 25 (blue tiles) ranks. We discovered clear patterns of abundance that were distinct for different groups and even subgroups of organisms. As seen along the left-most columns of the heat map (Fig. 2, black tiles at A1-K1 on the grid), a few SSRs are highly abundant across most organisms - viz. C, AC, AG and the polyA repeat classes including A, A(n)T/G/C (density > 100 bp/Mb). But they are rare in green algae and some of the fungi of the ascomycetes and basidiomycetes groups; some of these SSRs are in fact entirely missing in these groups as indicated by the red tiles (frequency < 10; B1 and B2 on the grid in Fig. 2).

Fungi instead have highly abundant ACG, CCG and other GC-rich repeats (black/blue tiles at B8-E8) that are not very abundant in multicellular organisms. Green algae show a high abundance of some GC-rich repeats (B8, B18, B20) correlating with their high average genomic GC content of 61.3%. Interestingly, in many fungi, especially from the Ascomycetes and Microsporidia subgroups, upto 95% of the repeat classes appear to be missing (red tiles at D7-D20 and E2-E20, respectively; frequency of occurrence < 10) though this trend shows a sharp change in the Basidiomycetes subgroup where only a few SSR classes are absent. Some of the Protists (row F, red tiles) and green algae (B2-B7, B9-B17) too show a similar trend. Most other organisms (486/719 organisms) have some representation of all 501 classes of SSRs. Notably, the ACGCGT repeat is absent in about 64% of the organisms across evolution (A10.5 – G10.5 and H10.5 – K10.5, red arrow), except for bees, ants, wasps, and some fungi. Similarly, AGCGCT (red arrow) also appears to be unpopular, missing from about 51% of the genomes, including all deuterostomes. Both these repeats in fact have the smallest lengths of occurrence as well (see below) suggesting that they are not well tolerated in genomes.

We next looked for SSRs that were highly abundant in only specific species or subgroups but not in any of the other organisms (Table 1). For example, a small group of land plants - cereals like maize, sorghum, millet, rice and corn (A8, arrow) - show some GC-rich repeats to be uniquely enriched. They also harbor abundant ACG and CCG repeats (A8) otherwise seen in fungi. Unique species-specific enrichment signatures can also be found in the *Leishmania* (F7) and *Drosophila* (G8, arrow) species as well as in higher organisms such as birds (I4), ruminants (bison, cattle, water buffalo, yak, sheep and goats - J5, J6, J14), and in primates (K6, AATGG and K9, ACCTCC, arrows) (Table 1 and Additional file 2: Figure S3). We also notice other subtle trends - for instance crocodilians such as gharials, crocodiles and alligators show a relative abundance for AGCCCC (I16) - but many of these are not uniquely abundant or significantly enriched specifically in their target species (*p* > 0.05) and are hence not analyzed further. Notably, the enrichment trend for specific SSRs is sharply contained within each of these groups of organisms (Fig. 2), clearly defining them as species-specific enrichment signatures. A few of these SSRs have also been previously identified as being enriched in a species-specific manner in smaller scale studies limited to a few species or subgroups [21–23].

SSR rank is calculated based on relative density compared to other SSRs within the organism as described in Fig. 4. An SSR is considered abundant in the species if it falls within the top 10 ranks. It is considered uniquely abundant (enriched) in a species if it falls within the top 10 ranks of SSRs in that species but not in any other species (*p < 0.05, **p < 0.001, ***p < 1E-05, t-test). The estimated divergence time point from the common ancestor is retrieved from Timetree (http://www.timetree.org), Ma = Million Years Ago.

### Length ranges of SSRs

We looked at the length of each SSR across all occurrences in the 719 organisms. As expected, longer SSRs were found to be present in the larger genomes (Spearman, r = 0.87; Additional file 2: Figure S4A). We identified the longest perfect SSRs found in each of the 719 organisms; AACCCT, the known telomeric repeat, is the longest SSR in 9% of the organisms (70 out of 743), with a top length of 15 kb in the fish *Rhincodon typus* (whale shark). We next looked at other longest repeats apart from the telomeric repeat and found that AT and AAT also frequently appear as the longest SSRs (in 8% organisms; top lengths 17.3 kb and 19.3 kb, respectively). The longest SSR seen among all organisms is almost 52 kb long – 12,980 perfectly repeating units of AAAT in the mammalian *Cercocebus atys* mapping to an intergenic region, at a distance of 23.5 kb from the nearest gene, LOC105598351.

**Fig. 3:**
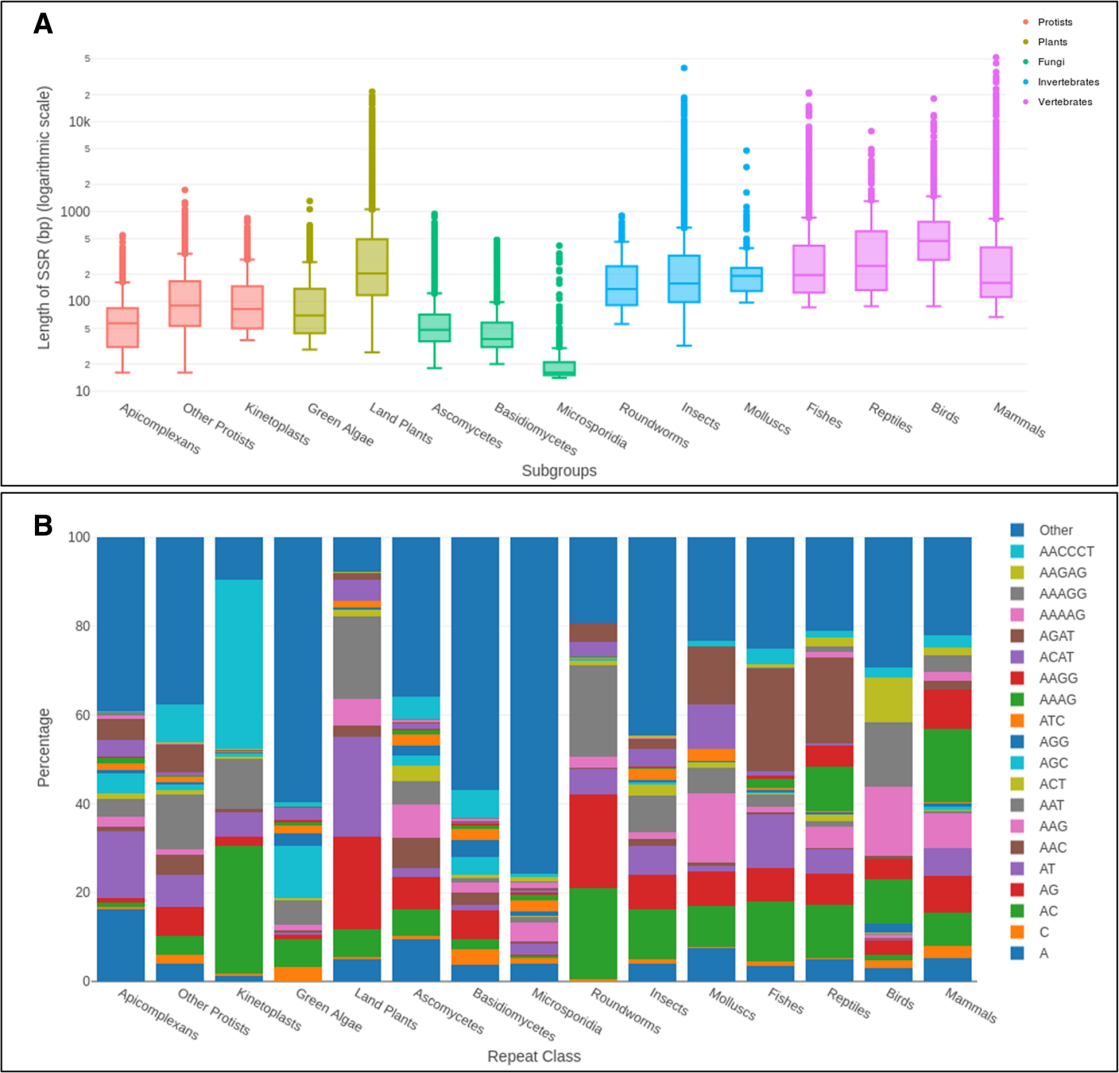
SSR lengths across subgroups. **a** The top 100 longest repeat instances from each organism are recorded separately and the data for organisms falling in the same subgroup are then grouped. Box plots showing the range of the longest 100 repeat lengths in the organisms of a particular subgroup are plotted. Color indicates the main group to which the subgroup belongs. **b** The percentage of occurrence of a repeat class in the top 100 longest SSRs across all organisms is calculated to select SSRs that occurred at least 1% of the times. The average percentage of these ‘highest occurring’ SSRs across organisms in each subgroup is plotted (Y-axis), categorizing the rest of the SSRs into “Other”. Individual SSRs are color-coded as indicated. The 15 subgroups are labeled along the X-axis

We then analyzed the length distribution for each SSR in a subgroup-specific manner (Fig. 3a). As a single instance of a long repeat could potentially be an outlier or an assembly error, and may not accurately reflect the general trend, we derived the 100 longest repeat instances from each organism, and grouped them by the subgroup of the organism. Figure 3a depicts the range of subgroup-wise longest instances. Differences among subgroups within a group could often be correlated with the differences in their genome sizes. However, birds are an exception to this: despite having genome sizes significantly smaller than reptiles (*p* < 10e-9, pairwise t test, Bonferroni corrected) and mammals (*p* < 2e-16, pairwise t test, Bonferroni corrected), their SSR length range is higher than reptiles and mammals (*p* < 2e-16, pairwise t test, Bonferroni corrected).

We further checked if the longest instances of repeats in a subgroup belonged to specific repeat classes. We observed that distinct SSRs preferentially exist as long repeating units in different subgroups (Fig. 3b). AACCCT, the telomeric repeat, is surprisingly not the longest repeat to be maximally represented in any subgroup except kinetoplasts (*p* < 10e-5, Fisher’s exact test). Instead, AGAT is the predominantly represented long SSR in fishes and reptiles, and AAAG in mammals. Dimers of AC, AG and AT vary in their representation as long stretches in different subgroups; plants have maximal representation of AG and AT as longest SSRs while animals generally have longer AC stretches than AG or AT stretches (p < 10e-5, Fisher’s exact test). We next calculated the median from the 1000 longest instances of each SSR class to check for the preference of various SSR classes to exist as long repeats (Additional file 3: Table S2). Interestingly, the median values range from as high as 1.26 kb (AT repeats) to as low as 15 bp (AGCGCT repeats). While no obvious common motif pattern could be observed for the classes that showed the longest lengths, SSRs which showed the shortest lengths appeared to be of motifs with GC-rich centers flanked by A/T, but never completely GC-rich (Additional file 2: Figure S4B), such as the two rarest repeats described in the previous section, AGCGCT and ACGCGT.

The frequency of an SSR is expected to decline with increasing repeat length, as longer repeats have a higher chance of being mutated. We have demonstrated earlier that certain SSRs, however, show a preference for occurring at higher lengths in some organisms [24], with greater protein binding efficiency at the preferred lengths [14]. A length preference is defined as a sudden increase in the frequency of occurrence seen at a particular range of SSR length (Additional file 2: Figure S5A). Our earlier work has indicated that 45 bp repeat size is the optimum length for a majority of the SSRs, especially in the human genome [24]. Here we confirm that length preference is seen for relatively longer SSR size ranges in all genomes (~ 50 bp), except in fungi where SSR preferred lengths are generally smaller (~ 20 bp) (Additional file 4: Table S3). We find that only 131 out of the 501 SSRs can be associated with a specific length preference in at least one organism. We tabulated the length preference of each of the 131 SSRs across all 719 organisms and converted it to a heat map of percentage of organisms within a subgroup that show length preference for a given SSR (Additional file 2: Figure S6). Fungi do not exhibit length preference for many SSR classes; none of the microsporidia show a length preference for any SSR (0%, red cells) while just 10–12% of Basidiomycetes show a preference for the polyA and polyC SSRs. In fact, as seen in Additional file 2: Figure S6, most unicellular organisms show no length preference for any SSR class. However, with increasing complexity of the organisms, a higher number of SSR motifs have accumulated as long repeats, the most being in mammals where as many as 85 SSR classes show a length preference (last column). This suggests that certain SSR motifs have been selected at long sizes for possible functional roles. AAAG and AGAT show a length preference in > 80% of mammalian species, followed by polyA and AAGG (73% mammals show length preference). This trend does not appear to be a function of the genome size (Additional file 2: Figure S5B).

### Genomic patterns of SSRs categorized by motif size

**Fig. 4:**
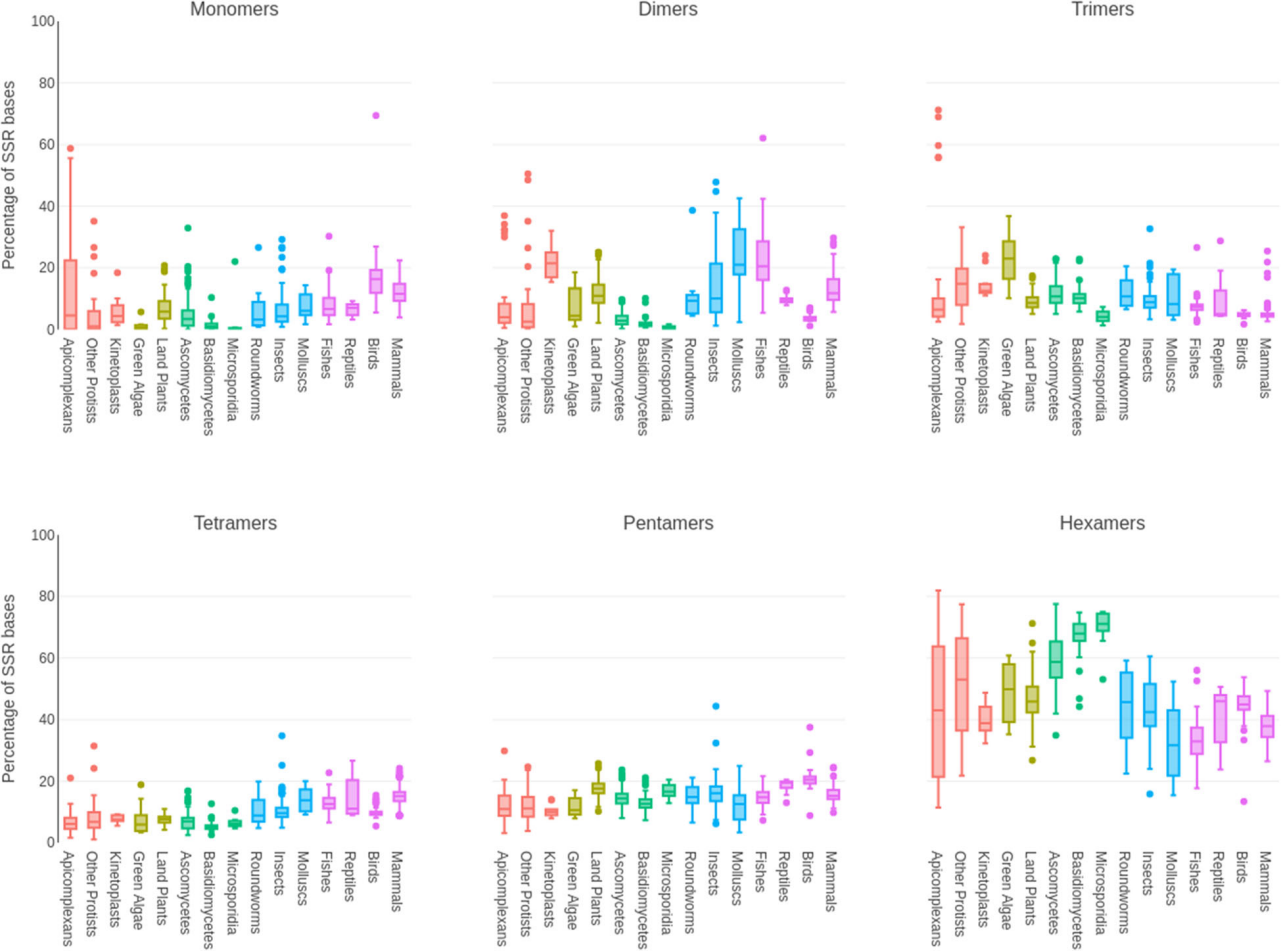
Composition of SSRs by their motif sizes. The number of bases covered by repeats of different motif sizes is summed across all organisms within a subgroup and divided by the total number of bases covered by all SSRs in that subgroup. Box plots for each subgroup represent the percentage of each k-mer base coverage (Y-axis) in the subgroup (X-axis)

To check if different repeat classes are preferred through evolution, we looked at the distribution of SSRs across all the subgroups based on the size of the base repeat motif (Fig. 4). We categorized the repeat classes as monomers to hexamers for repeats with motif sizes of 1 to 6 nt. We find that monomers are abundant in birds and mammals (15–17% of total SSRs) and rare in green algae and fungi. Within mammals, primates have the highest contribution of monomers (18%; Additional file 2: Figure S7A). Of the two possible monomers, polyA is largely preferred by all subgroups other than green algae (Additional file 2: Figure S7B), which is a reflection of their genomic GC content. The polyA bias is conspicuous especially in mammals, where A contributes > 90% of the monomers as seen in Additional file 2: Figure S7B; in primates, the proportion of polyA rises to 99% of all monomers (Additional file 2: Figure S8A). Birds and fungi have the lowest dimer content (Fig. 4) while molluscs and fishes have the highest (median % = 20.93 and 20.52, respectively). Insects, mammals and land plants show similar abundance (~ 11%) but within mammals, rodents are prominent in their high dimer content (17.96% in rodents vs 11.8% in other mammals; Additional file 2: Figure S7A). CG repeats are extremely rare, contributing to less than 1% of all dimers in most species except green algae and basidiomycetes, where they contribute to 9.5 and 6% of dimers, respectively (Additional file 2: Figure S8B). AT repeats constitute the highest share of dimers in apicomplexans (93% of dimers) and land plants (62.3% of dimers), while AC dimers are the most frequent dimers in species of most other subgroups. Similar trends in dimer abundance have been previously documented for a few vertebrate species [3].

Trimers are especially low in proportion in higher vertebrates such as birds and mammals but in green algae and some protists, trimers constitute a large proportion of all motif types, second only to their hexamer content (Fig. 4). Overall, hexamers are the predominant SSR type in all organisms. Their proportion however is lower in complex organisms compared to protists, plants and fungi where they are the most abundant (~ 70% in microsporidia). Tetramers too show a noticeable difference, but in the opposite direction - tetramer percentages are lower in simpler organisms (protists to fungi) compared to protostomes and deuterostomes. Tetra- and pentamers show least variance in distribution across subgroups while dimers show the maximum divergence. These trends highlight the differences in the organization of eukaryotic genomes and could be a reflection of the variable SSR mutation rates and propagation among the coding and non-coding compartments.

### SSR distribution in genomic features

**Fig. 5:**
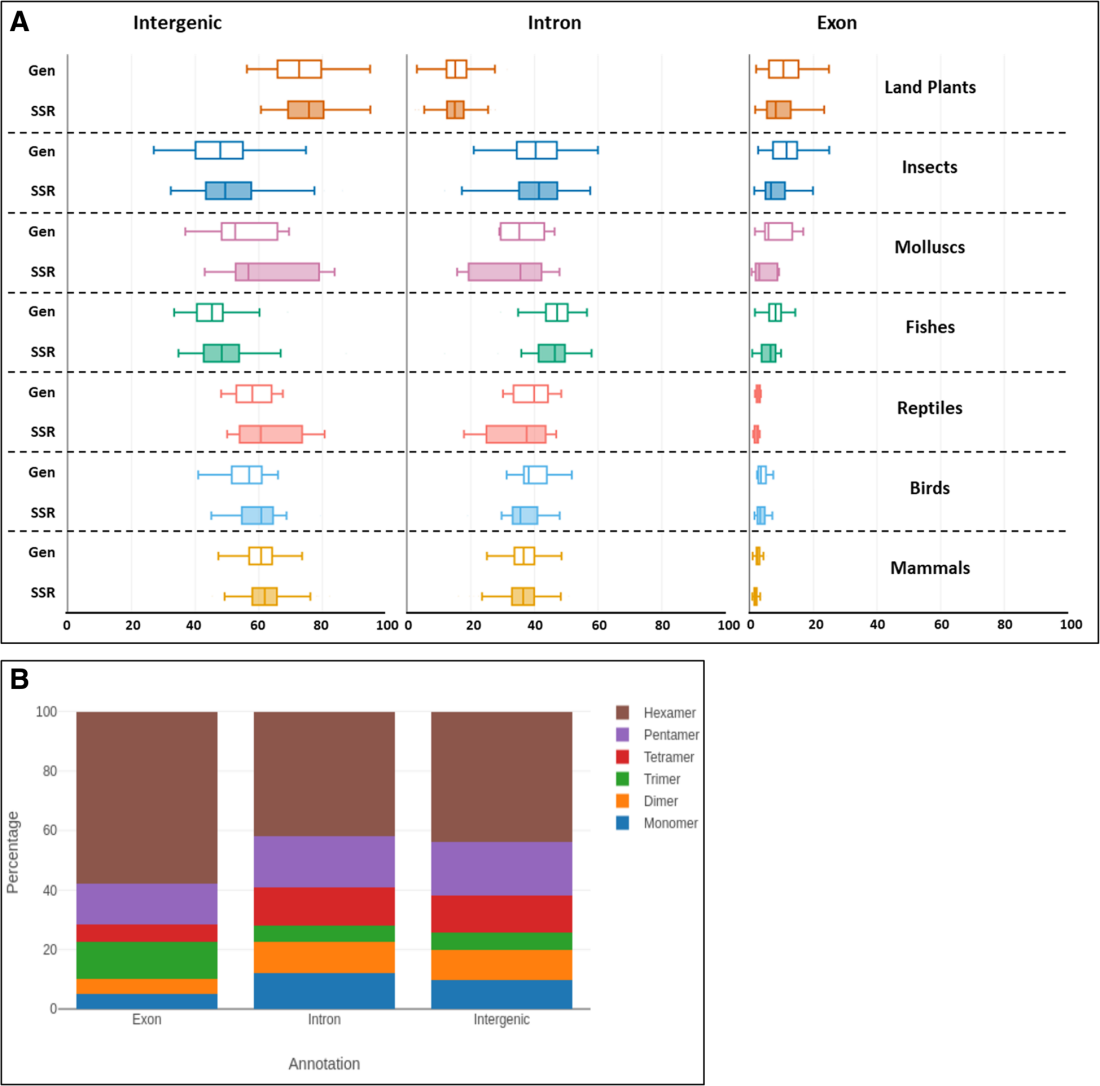
Motif size dependent distribution of SSRs among various genomic features. **a** Boxplots representing the proportion of SSRs in intergenic regions, introns, and exons across subgroups. The distribution of SSRs in intergenic and intronic regions (shaded boxes) mostly mirror their genomic distributions (non-shaded boxes). However, we see a small but significant underrepresentation of SSRs in exons (right panel, *p* < 10e-40, paired-sample t-test). **b** Average distribution of SSRs in coding and non-coding regions in all species studied, colored by the size of repeat motif. The percentage of trimer and hexamer repeats is higher in exonic SSRs at the expense of tetramers and dimers. The fraction of each motif size is calculated as the total bases covered by SSRs of a given motif size divided by the total bases covered by all SSRs. This value is multiplied by 100 to derive percentages (the total for all motif sizes add up to 100), represented on the Y-axis

There has long been evidence for non-random genomic distribution of SSRs showing differential distribution across chromosomes and genomic features [1]. We therefore looked at the distribution of SSRs across 334 organisms spanning 7 of the 15 subgroups for which genome annotations are available (Additional file 1: Table S1) in order to understand biases in distribution trends and possible functions. Overall, the distribution of SSRs in intergenic, intronic and exonic regions reflects their genomic distribution with intergenic regions having the highest abundance of SSRs (Fig. 5a). However, we do see a small but significant underrepresentation of SSRs in exons (*p* < 10e-40, paired-sample t-test). This distribution remains the same across all the 7 subgroups, correlating with the respective genomic percentages of intergenic regions, introns and exons. We checked for specific differences in the genomic distribution of SSRs with respect to the repeat motif size. Introns and intergenic regions mostly show a similar distribution except in monomers which are slightly enriched in introns (Fig. 5b). Exons, however, show a significant difference in the proportions of different motif sizes compared to the non-coding regions. Trimers and hexamers increase in exons compared to introns by ~ 7 and 16%, respectively (Fig. 5b). An earlier study has indicated that trimers are the most abundant group in exons [3], but we find that in fact hexamers tend to be more abundant in exons, similar to the trend observed in non-coding regions. Dimers and tetramers are under-represented in exons as expected due to the disruption they can cause to the coding frame, occurring at higher frequencies in non-coding regions.

Subgroup specific motif size distributions across genome annotations replicate these broad trends between exonic and non-coding regions (Additional file 2: Figure S9). Most repeat types such as monomers, dimers and tetramers are generally more abundant in introns and intergenic regions compared to exons across all taxa, while trimers and hexamers increase in exons. Mammals show an abundance of tetramers compared to trimers in introns and intergenic regions, a trend mirrored in other deuterostomes albeit to a lesser extent. But the same is not seen in land plants and they have equivalent tetra- and trimers in their non-coding regions. Dimers are under-represented in exons across all groups, occurring at higher frequencies in non-coding regions. Interestingly, dimers are most enriched in the non-coding regions of fishes and molluscs contributing to 18–27% of SSRs, comparable to their hexamer content, while birds have the lowest contribution of dimers across all genomic regions (2–3%) as mentioned earlier.

## Discussion

Microsatellites are increasingly being recognized as critical sequence components with multiple roles in genome regulation. We identified perfect SSRs of 1–6 nt motifs from 719 genomes spanning 15 eukaryotic taxonomic subgroups including protists, plants, fungi, invertebrates and vertebrates. We find that the distribution pattern of SSRs is a characteristic of the species or subgroup of the organism and that different taxonomic groups have distinct patterns of microsatellite presence, abundance and length preferences.

Longer repeats are generally found in larger genomes and most repeats tend to decrease in frequency at higher lengths. PolyA-rich repeats such as AT and AAT and A(n)G tend to have the longest lengths of occurrence. Earlier studies [3] have shown dimers to be the predominant longer repeats in introns and intergenic regions of genomes (except fungi). Subgroup specific longest SSRs identified in this work include AGAT (fish, reptiles) and AAAG repeats (mammals) as the predominant long repeats in vertebrates while AG/AC/AT dimers and AAT are frequent long repeats in lower organisms and in land plants (AT/AG). 131 out of the 501 possible repeat classes show a specific enrichment at longer lengths in at least one species studied; mammals display a length preference in 65% of these repeat classes while none of the fungi and only some protists show a length preference in any repeats. The preferred accumulation of longer repeats may point to a selection pressure on these elements in a repeat class- and organism-specific manner. We have earlier shown that AGAT repeats show a preferred length of 40-48 nt (10–12 repeat units), and these elements function as enhancer blockers in *Drosophila* and human cells [14]. In this context, we find that AGAT shows a length preference in many species (171 out of 719, out of which 82 are mammals), suggesting that its functional role could have been co-opted by multiple organisms.

The sharp boundaries of change in SSR abundance coincide perfectly with the phylogeny of groups and subgroups. We were able to identify many taxon-specific microsatellite patterns within closely related species. In the light of the high polymorphism of microsatellites normally seen among individuals of the same species, identification of tightly conserved species-specific enrichment is useful in the context of microsatellite markers. Whether specific enrichment of a particular SSR indicates functional significance or mirrors close evolutionary relationships remains to be verified experimentally. Repeat elements have long been thought to have functional, mutational and evolutionary significance [25, 26] and experimental evidence of a role for SSRs is increasingly available, albeit in only a few species per study (reviewed in [27]). While this manuscript was being reviewed, another study was published that showed differential distribution of human-specific SSRs at translation initiation sites [28] with implications for directing species-specific translation events and thus the phenotype. Such studies bolster recent work on the evolutionary trends and influence of SSRs in defining species specificity and warrant larger scale analysis across the evolutionary landscape.

**Fig. 6:**
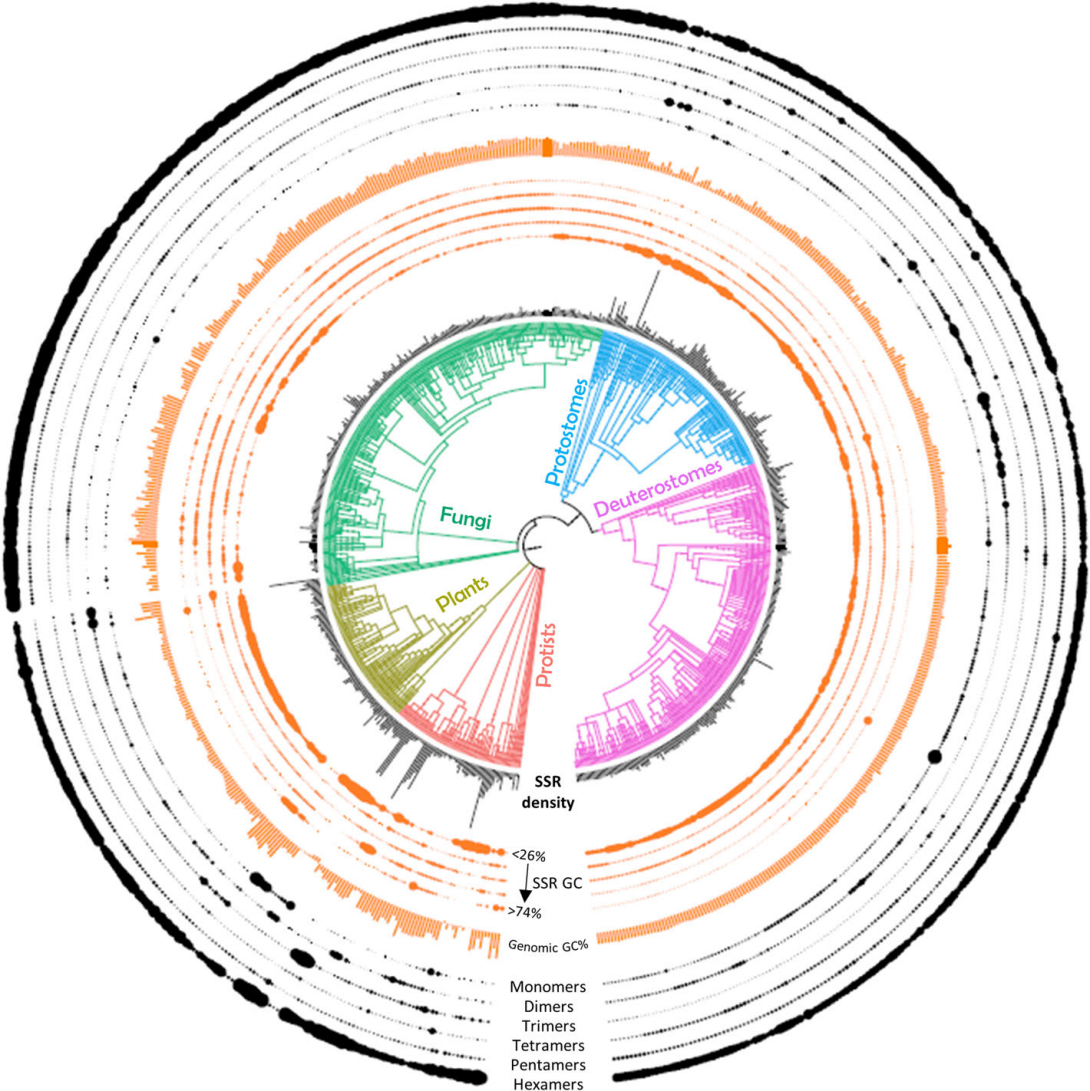
Phylogenetic tree representation summarizing attributes of all SSRs analyzed. The tree was constructed using iTOL (interactive Tree Of Life) webserver. The clade nodes are colored based on the 5 groups used in this study. Black bars (the innermost track) around the organisms represent the SSR density (bases covered per MB of genome) in each organism. The orange tracks around the SSR density show the SSR GC% in each organism (the innermost orange track represents the relative enrichment of motifs with <=25% GC, while the outermost orange track represents SSR GC > =75%) and the middle three tracks represent intermediate GC ranges. The size of each dot on the track (representing each organism) indicates the amount of SSRs present in that GC range. The orange bars represent the genomic GC content. The black tracks show the distribution in each organism based on the motif size of the repeat (the innermost black track represents monomers while the outermost black track represents hexamers). The size of each dot on the track (representing each organism) indicates the proportion of SSRs present in that motif size range

Interestingly, most fungi do not have > 90% of the SSR classes, and also show the lowest SSR densities, indicating that these species may be under a different set of constraints in what their genomes can adopt. Other lower organisms – such as some protists and green algae – also lack many repeat classes while invertebrates and vertebrates have a representation of almost all SSR classes. Exceptions are the ACGCGT and AGCGCT repeats that occur rarely in evolution, with > 50% of all organisms excluding them altogether, including many vertebrates. Even in the few species of hymenopterans such as ants, bees and wasps where these repeats occur at a moderate frequency, they are of short lengths. We hypothesize that such sequences constitute a rare class of SSRs that are not tolerated in genomes. We find large variations in the densities of SSRs in protists, a subgroup that encompasses both SSR-dense as well as SSR-sparse genomes. More complex organisms, however, show lesser variation in SSR densities (summarized in Fig. 6), suggesting greater constraints operating upon their genomes. Protists have very varied SSR GC contents as well - *Dictyostelium* and *Plasmodium* species harbor AT-rich SSRs while *Eimeria* and *Micromonas* species have GC-rich repeats. Green algae have a preponderance of GC-rich SSRs while most fungal SSRs are of intermediate GC content. On the other hand, complex genomes, including in land plants, carry only AT-rich SSRs with most GC-rich repeats having been filtered out (Fig. 6). This stability in SSR composition is reflected in the relatively uniform content of genomic GC seen across vertebrates. We found abnormally high enrichment of select repeat classes in protists, viz. A repeats in *Plasmodium* species and AGC repeats in *Eimeria* species, which contribute to 40–70% of their total SSR content. An interesting coincidence is that most of these protists are parasites. It needs to be further explored whether the preferential enrichment of a single repeat type in their genomes is beneficial to their pathogenesis.

The integrity of genome assembly and sequence information for different organisms can be an issue in such analysis, affecting SSR identification and inferences. In this context, our analysis of trends across subgroups alleviates, to an extent, the problems arising due to poor quality information from a particular genome; outlier trends need to be examined on a case by case basis as they might reflect the quality – accuracy and completeness - of the genome assembly. While this work provides a global framework for understanding microsatellite function, it is important to understand the role(s) of specific SSRs in the context of their genomic locations. However, the current level of genome annotation does not allow such an analysis at this scale, limiting this study to the analysis of SSR distribution trends in exonic, intronic and intergenic regions for 334 genomes where such annotations are available. The observations presented in this work serve as a snapshot of eukaryotic genomes in the context of perfect SSRs – instances of repeats that were preserved without even a single nucleotide change. We have not considered imperfect SSRs in our study to avoid limitations posed by currently available imperfect SSR identification tools. Hence, this work cannot address whether these elements are a consequence of DNA repair / replication errors or mutational mechanisms. Different taxonomic groups have distinct patterns of microsatellite presence and abundance, representing their establishment in a common ancient founding member of all organisms in the group. Evolutionary trends thus correlate with the preferential and selective inclusion of SSRs that may have been retained for the advantage they confer via novel regulatory mechanisms. These are testable hypotheses and the SSR signatures identified in this work can serve as a starting point for understanding this paradigm.

## Conclusions

We have identified nearly 685 million perfect SSRs across 719 eukaryotes to reveal constraints in SSR distribution across evolutionarily related species. Our results provide a comprehensive framework to understand microsatellite features in genomes. We have documented novel species- and subgroup-specific patterns of SSR enrichment that offer insights into microsatellite conservation among taxonomic groups. Interestingly, some defined patterns seem to persist in genomes, especially across multicellular and complex organisms, suggesting a role for SSRs in the genome regulatory toolkit.

## Methods

### Data collection

The latest versions of eukaryotic genome sequences available on NCBI’s RefSeq database were downloaded from the FTP site of NCBI (ftp://ftp.ncbi.nlm.nih.gov/genome). The primary classification of each organism was adopted from NCBI using the hierarchical classification of kingdom, group, and subgroup. As our study was limited to eukaryotic species, all organisms belonged to the kingdom “Eukaryota”; the group Animalia was subdivided into Protostomes and Deuterostomes. In order to investigate globally conserved evolutionary trends, we utilized the subgroup information of NCBI to categorize all the species. Poorly represented subgroups (< 5 species) were removed from the analysis to avoid sampling error; these included 2 poriferans, 3 cnidarians, 3 flatworms, 4 arachnids and a few singleton species belonging to distant taxa. We preferred not to merge clades containing few representatives as this could confound the trends in favor of the most represented sub-clade. The final list consisted of 719 species spanning 15 subgroups (Fig. 1 and Additional file 1: Table S1). Detailed taxonomic classification of these organisms was gathered using an R package *taxize* [29]*,* which fetches the class, order and family information of species where available. All the organisms were arranged in an evolutionary order using TimeTree [30] and this order was consistently followed for all further analyses. The hierarchical classification provided by TimeTree was downloaded as a newick file and was used for visualization using the iTOL (interactive Tree of Life) webserver [31].

### Identification of SSRs

Perfect SSRs > = 12 nt in length were identified from sequences of all downloaded genomes using a Python-based exhaustive algorithm, PERF [15]. We chose to work with SSRs that were at least 12 nt in length in order to consider at least 2 complete repeating units of hexamers (the largest motif size in the study). The 5356 possible permutations of 1–6 nt long DNA motifs were grouped into 501 unique classes of SSRs based on the cyclical variations and strand of the motif sequence, as described previously [32]. A repeat class motif represents all the motifs which are cyclical variations of itself and of its reverse complement (Additional file 5: Table S4). PERF reports all SSR locations in the genome in BED format, with additional columns describing the length of the repeat sequence, the repeat class, number of times the motif is repeated in tandem (repeating units), and the actual repeat motif (defined by the start of the SSR sequence, irrespective of repeat class). Using these parameters, we identified a total of 684,885,656 repeats from the genome data of 719 species.

### Calculation of basic SSR attributes

For each organism, we calculated a few parameters that outline the prevalence of SSRs in the genome. SSR frequency is the total number of SSRs found in the genome. The total bases covered by SSRs in the genome is calculated by summing the lengths of all the SSRs. To normalize for differences in genome sizes across evolutionary groups, we derived the SSR density for each genome, defined as the number of bases covered by SSRs per MB of genome. This was calculated by dividing the total SSR bases with the genome size in MB. We have used SSR density for comparisons throughout the study, unless otherwise mentioned. The SSR GC% of an organism is the GC% of the sequence formed by concatenating all the repeat sequences found in the genome. A master table containing all the SSR attributes, along with the taxonomical classification and genome information of each organism, is available in Additional file 1: Table S1, Sheet 1.

### Repeat class specific abundance trends across evolution

SSR frequency, base coverage, and density for each of the 501 repeat classes were calculated in each organism using in-house Python scripts. To identify repeats that are enriched/absent in various sets of organisms independent of their taxa, we ranked all the repeat classes based on their density in each organism. Briefly, we first gave the lowest score of − 2 to those repeats which had a frequency of < 10 in a given organism, to reduce sampling bias. Further, we assigned scores 3, 2, and 1 to repeats with the top 10, 25 and 100 ranks in the genome, respectively. Repeats in the bottom 100 ranks and frequency of at least 10 were given a score of − 1. All other repeats were assigned a score of 0. A matrix was built using the score information, where each row represents an organism and columns represent the repeat classes. Hierarchical clustering of the repeat classes was done using the Euclidean distance between columns of the matrix. This scoring system was used to maximize the clustering of repeats based on similarities in enrichment/absence, as Euclidean distance is sensitive to variance in the data. The clustered matrix was visualized as a heatmap using Morpheus (https://software.broadinstitute.org/morpheus/), an interactive tool for generation and exploratory analysis. The color scale on the heatmap ranged from a high score of 3 (black) to a lowest of − 2 (red) as described above and in Fig. 2. The repeats and organism information in the heatmap can be obtained by loading the provided json file into Morpheus (Additional file 6: Heatmap.json). The ranks were used for heatmap generation and clustering to identify clade-specific signatures. These signature motifs were further validated using statistical tests based on the original SSR density values (Table 1).

### Length preference analysis

Contrary to the expected gradual decrease in abundance of longer repeats, some repeat classes show an increase in abundance at longer lengths. This pattern appears as a peak with local maxima when unit length vs abundance for a repeat class is visualized as a line chart (Additional file 2: Figure S5A). A custom Python script was developed to detect repeat classes showing this pattern in all organisms (Additional file 7). Briefly, the script compares the abundance at consecutive unit lengths to detect an increase. The unit length before the first detected increase is considered the peak start. The script further checks if the increase continues to a local maxima followed by a decrease in abundance. The endpoint of the curve is defined as the unit length where the abundance goes lower than the abundance at the peak start. To filter false positives, we only considered instances which span at least 4 consecutive unit lengths (start and end included), and where the abundance at the peak start is greater than 10.

### SSR composition by motif size and GC%

Repeat classes were categorized based on the length of the base repeat motif as monomers to hexamers. A size category is defined as the group of repeat classes encompassing all SSR motifs of the same length. The base coverage of a size category is calculated by summing up the base coverage for all the repeat classes falling in that category. For GC composition analysis, we categorized the 501 repeat classes into 5 groups based on the GC content of the repeat motif. This was calculated using the 12 bp string (minimum length cutoff) formed by repeating the base motif in tandem. The 5 groups of SSR GC content are <=25%, 26–49, 50%, 51–74%, > = 75%, which encompass 70, 120, 133, 108, and 170 motifs respectively.

### Genomic annotation of SSRs

GFF files containing gene annotation information of various organisms were downloaded from the FTP site of NCBI (ftp://ftp.ncbi.nlm.nih.gov/genome). Annotation for SSRs was done based on the GFF files using an in-house Python script (Additional file 8). In brief, the script uses the genic and exon coordinates to identify SSRs that overlap either exons, introns, or intergenic regions. For each SSR, the output includes its genomic annotation, and its distance to the nearest TSS. In addition, for all exonic SSRs, the percentage overlap of SSR with an exon is reported. This was done to ensure that our results are not skewed because of a high proportion of SSRs falling within exon-intron boundaries. We verified that > 95% of exonic SSRs show a complete overlap with exons.

### Statistical analysis

Two sample t-test was done using t.test() function in R. Pairwise calculations were done using pairwise.t.test() in R, and *p*-values were adjusted using Bonferroni correction. Paired sample t-test was done using ttest_rel() function of SciPy package in Python. F-test to assay the significance in the variance of SSR densities was performed using var.test() function in R. One-way ANOVA (Analysis of variance) followed by Tukey’s post-hoc tests were done using aov() and TukeyHSD() functions of R respectively, using a confidence interval of 0.99. Fisher’s exact test was done using fisher.test() function of R. Plots were made using ggplot2 and the Plotly API of R and Python unless specified otherwise.

## Additional files

Additional file 1: **Table S1.** Mastersheet of SSR attributes and genomic information for 719 organisms arranged in evolutionary order. (XLSX 129 kb)

Additional file 2: **Figures. S1-S9.** Along with their legends. (PDF 1221 kb)

Additional file 3: **Table S2.** Table of SSR- and species-wise median lengths of longest repeat instances (XLSX 101 kb)

Additional file 4: **Table S3.** Table of length preferences (DOCX 13 kb)

Additional file 5: **Table S4.** Table explaining the classification of SSR motifs. Repeat classification is shown for a normal motif (AAG), a palindrome (ACGT) and a cyclical variation of a palindrome (AGCTCG, cyclical variation of CTCGAG) (XLSX 8 kb)

Additional file 6: Heatmap.json. Organisms and their SSR information, to be viewed as a heatmap in Morpheus (https://software.broadinstitute.org/morpheus/) (JSON 868 kb)

Additional file 7: LengthPreference.py. Python script for identifying SSRs showing length preference (PY 3 kb)

Additional file 8: GenomeAnnotation.py. Python script for genome annotation (PY 13 kb)

## Abbreviations

ANOVA: Analysis of variance
BED: Browser Extensible Data
FTP: File Transfer Protocol
GFF: General Feature Format
iTOL: interactive Tree of Life
SSRs: Simple Sequence Repeats
