# Supplementary material for: Patterns of microsatellite distribution across eukaryotic genomes
Source: BMC Genomics. 2019 Feb 22;20:153. doi: 10.1186/s12864-019-5516-5 (PMC6387519; doi:10.1186/s12864-019-5516-5)
Supplement: Supplementary file 2 — Figures. S1-S9. Along with their legends. (PDF 1221 kb) [file 12864_2019_5516_MOESM2_ESM.pdf]

Patterns of microsatellite distribution across eukaryotic genomes

Surabhi Srivastava, Akshay Kumar Avvaru, Divya Tej Sowpati, Rakesh K Mishra

Supplementary Figures

A

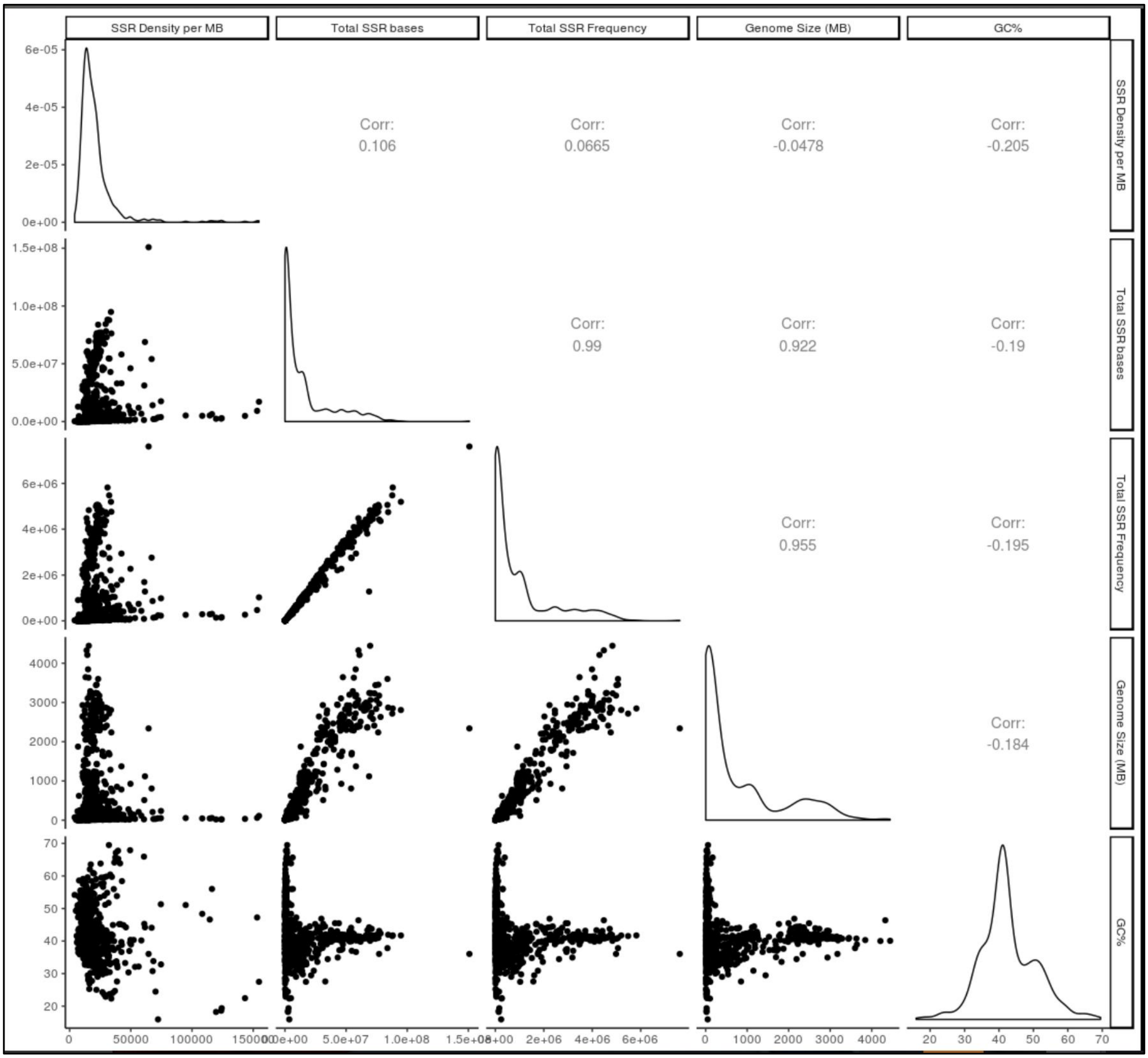

B

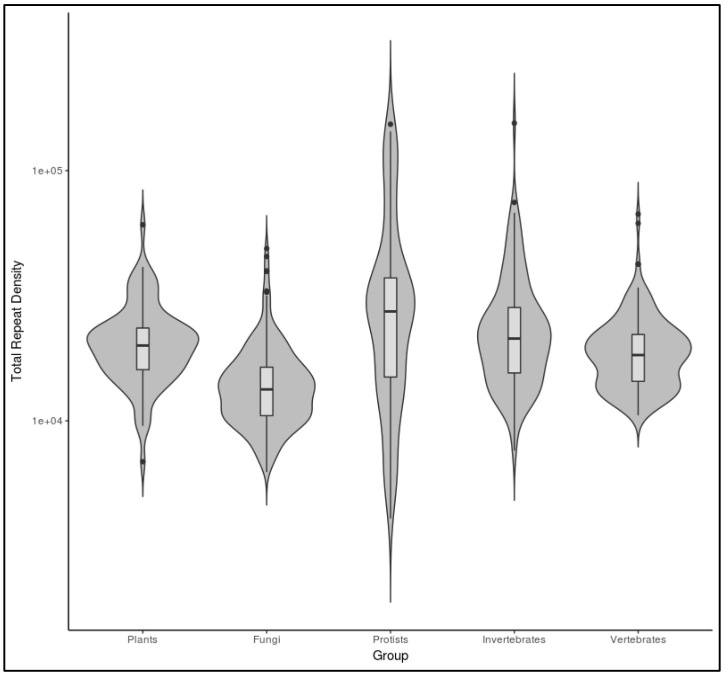

Figure S1: SSR attributes

A) Correlation matrix of SSR and genomic parameters.

The SSR density (bp covered by SSRs per Mb of genome, 1st column) is not correlated with the genome size or the genomic GC% (corr = -0.184 and -0.205, respectively). Abundance of SSRs (total SSR bases and SSR frequency, 2nd and 3rd columns respectively) is correlated with genomic size (corr = 0.922 and 0.955, respectively) but not with genomic GC% (corr: Pearson, r = -0.19). **B)** SSR density distribution across the 5 groups.

A

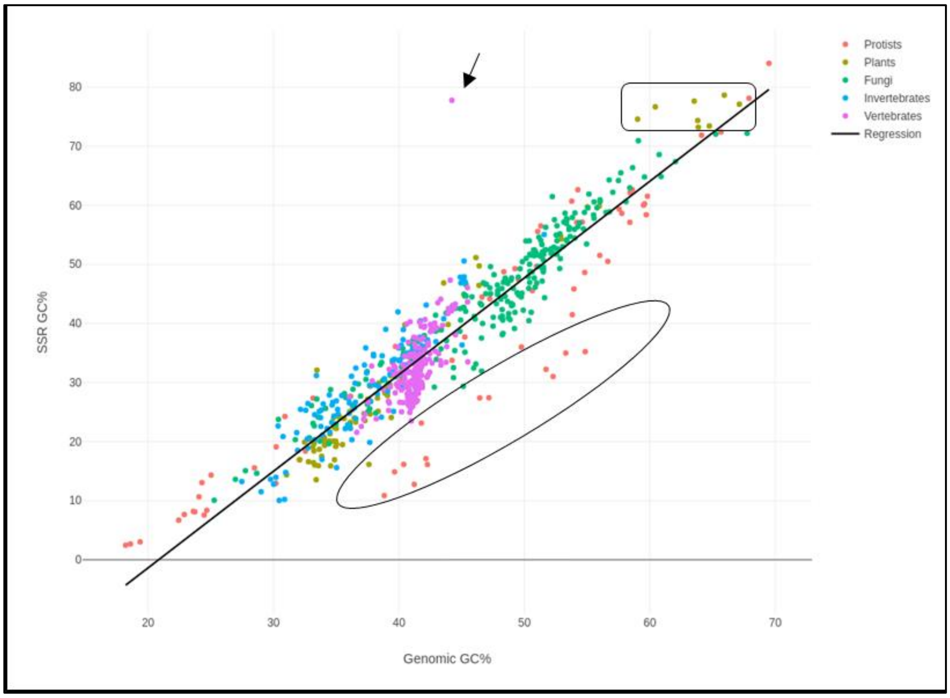

B

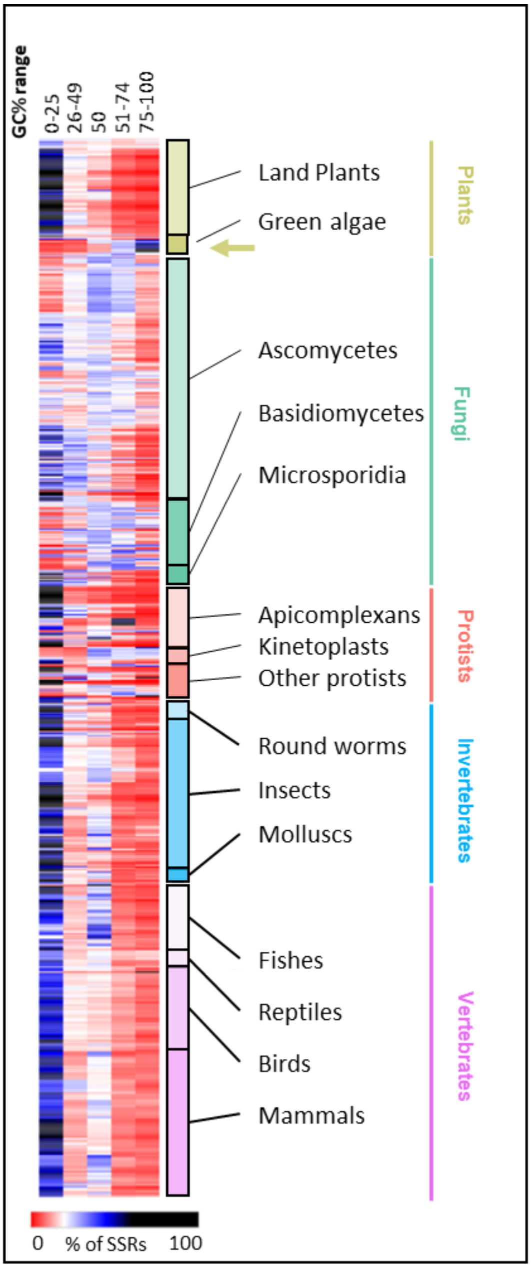

Figure S2: GC content of SSRs

A) Correlation between the GC content of SSRs and the genomic GC percentage. SSR GC content is obtained by concatenating all the SSR sequences in the genome and calculating their GC% (Y-axis). This correlates well with the genomic GC% (X-axis) for most organisms (Pearson, r = 0.94) other than a few outliers distant from the fitted regression line. For example, at 77.8% the collared flycatcher bird (*Ficedula albicollis*, arrow) has a uniquely high SSR GC content among vertebrates. Green algae also have high SSR GC% as a reflection of their GC rich genomes (boxed). Many protists, including some of the *Plasmodium* and *Trypanosoma* species (circled) show a slightly lower SSR GC content. The colors indicate the division of the organisms into the 5 main groups as per the legend on the right.

B) Subgroup specific patterns of GC content in SSRs

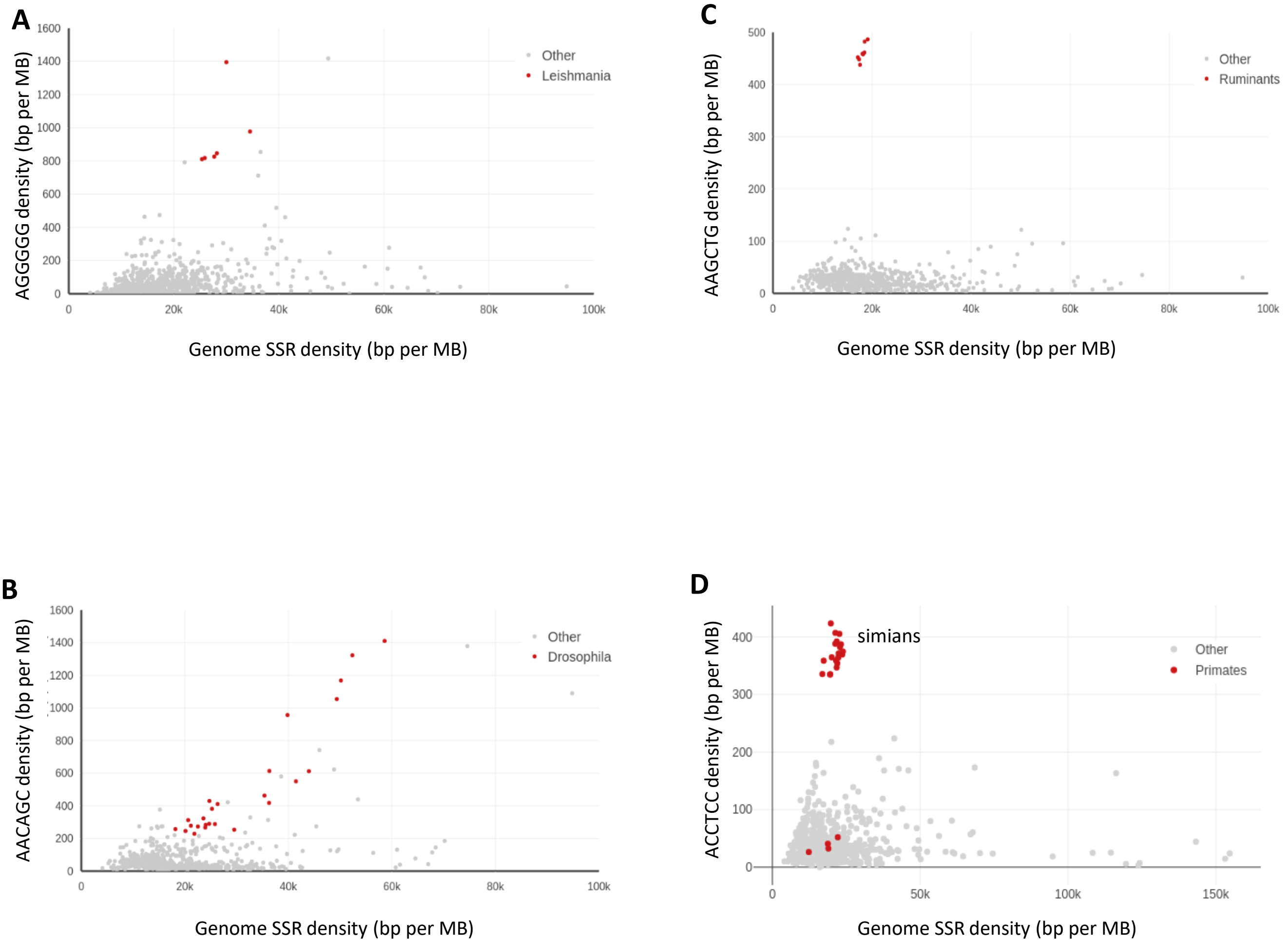

Figure S3: Enriched SSR signatures. Representative scatter plots of densities showing specific SSRs enriched in **A)** *Leishmania*, **B)** *Drosophila* and **C)** bovid species and **D)** Simians ($p < 0.05$) among all other 719 organisms

A

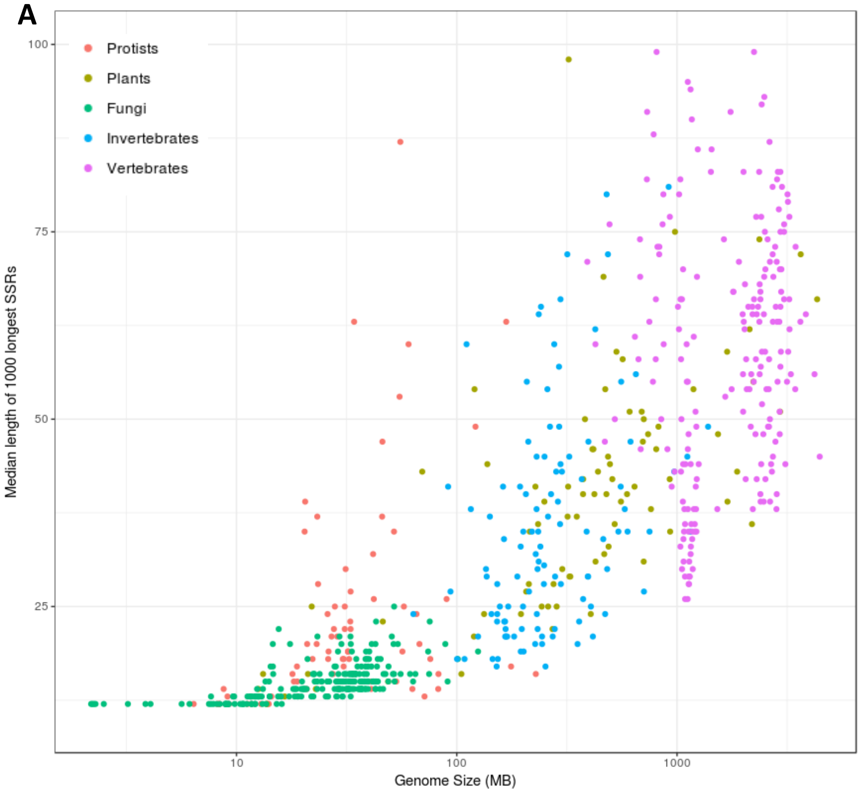

B

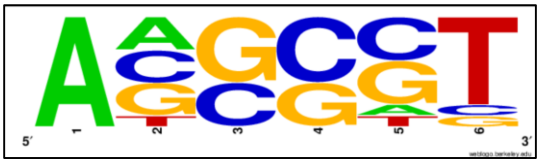

Figure S4: A) SSR lengths vs genome size
Scatterplot indicating the correlation between length of SSRs and the genome size of an organism (Spearman, r = 0.87). X-axis indicates the genome size in Mb on a log scale. Y-axis is the median length (bp) of the 1000 longest repeats present in an organism. The Y-axis is in log scale, and is trimmed to a maximum of 100 to remove a single outlier –Ficedula albicollis (collared flycatcher), which has a median length of 1897 bp. Colors of the dots indicate their group.

B) Consensus motif of SSRs that are always short (maximum median length = 18 bp).
The median lengths of the 1000 longest instances of each SSR across organisms was calculated, and the SSRs with 10 lowest medians were selected. The 10 SSR motifs with the lowest medians were used to create a consensus motif as a frequency plot of each base at a given position using WebLogo (http://weblogo.berkeley.edu/logo.cgi)

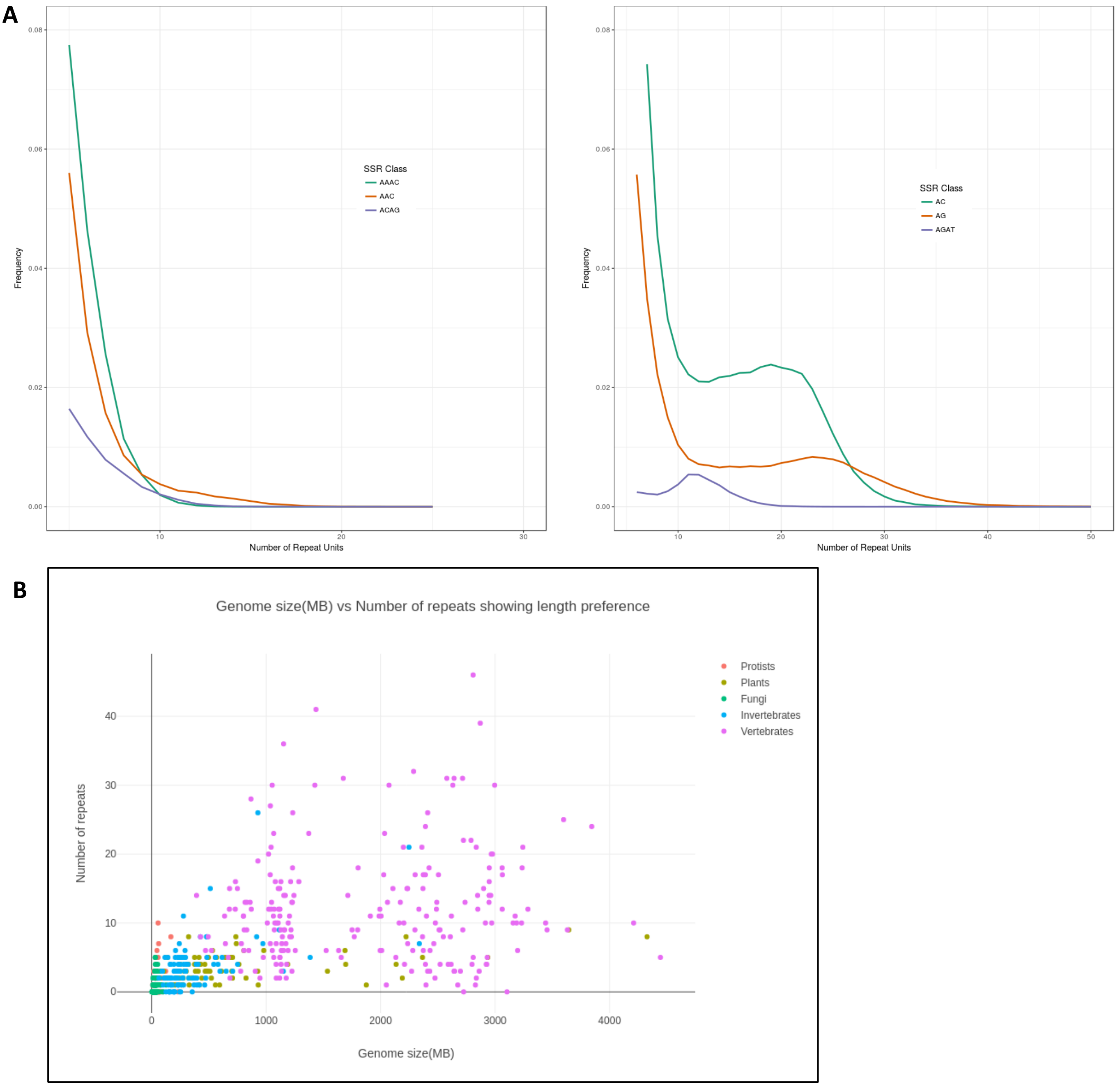

Figure S5: Length preference in SSRs

A) **Some SSRs show a length preference.** Line plots showing the preference of some repeats for enrichment at longer lengths. X-axis indicates the number of times the base motif is repeated, and Y-axis is the fraction of the repeats at the respective length. **Left panel**: AAAC, AAC, and ACAG repeats, which do not show a length preference. **Right panel:** AC, AG, and AGAT repeats, which show a bump indicating length preference at ~20, 25, and 12 repeating units respectively.

B) **Scatterplot of genome size vs number of repeat classes showing length preference.** Length preference indicates selective enrichment of a given SSR class at longer lengths, and is identified using a custom Python script (see Methods). The number of SSRs which showed a length preference in an organism is recorded and plotted on Y-axis, whereas the genome size of an organism in Mb is plotted on X-axis. The colors indicate the group of the organism. There appears to be no correlation between the genome size and the number of SSR classes that show a length preference.

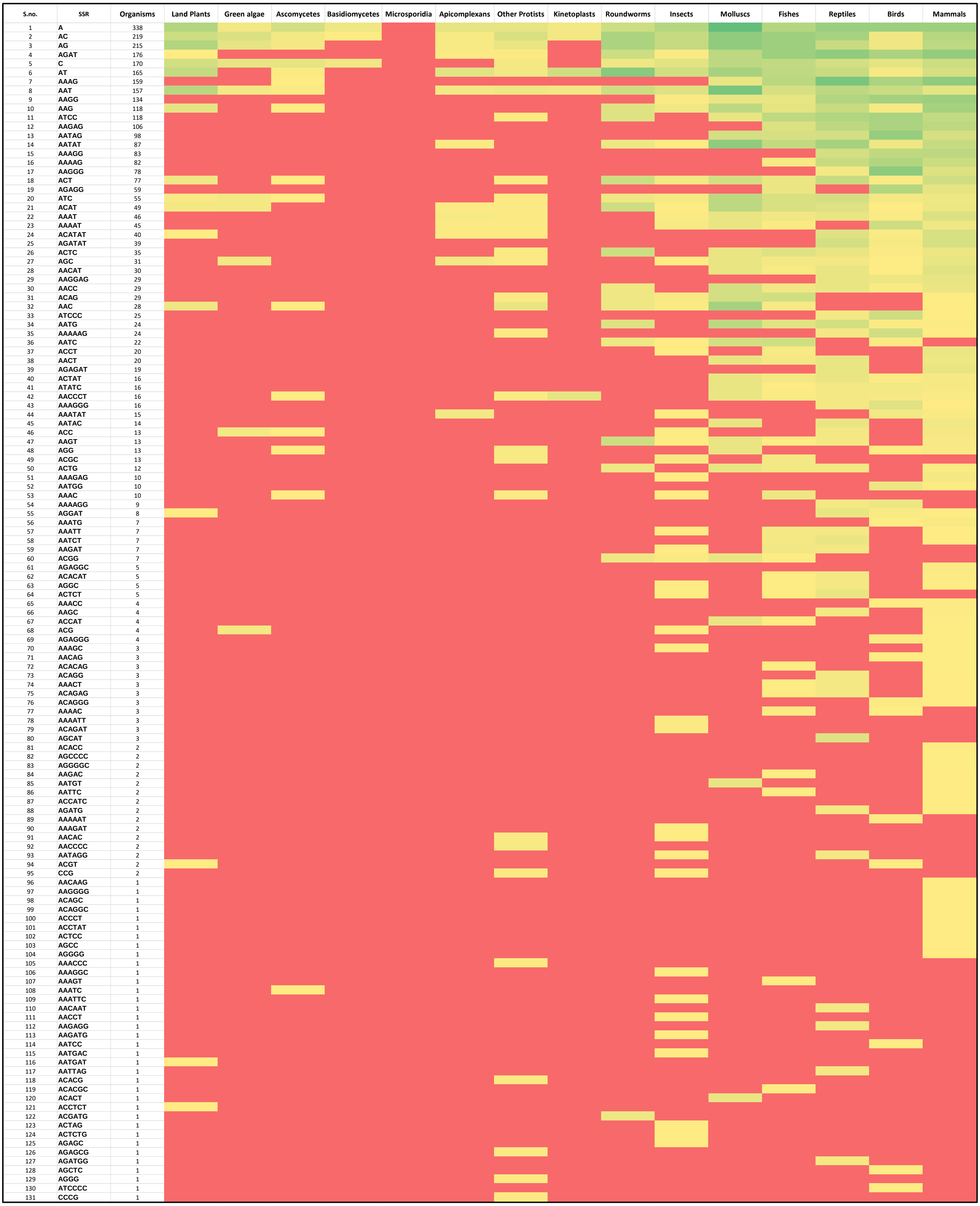

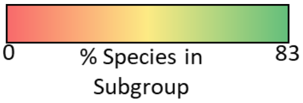

Figure S6: Summary of genomes that show SSR length preference

The 131 SSRs that show a length preference in any organism are arranged in rows. The number of organisms (totaled across all subgroups) that show a length preference for each SSR are indicated in the 3rd column. The column headers indicate the 15 subgroups. The percentage of organisms in each subgroup (maximum 83%) that show a length preference for each SSR is indicated as a heatmap. Complex organisms prefer specific longer repeat lengths, for example many mammals show a length preference for a majority of the SSRs (for 85 SSRs out of 131).

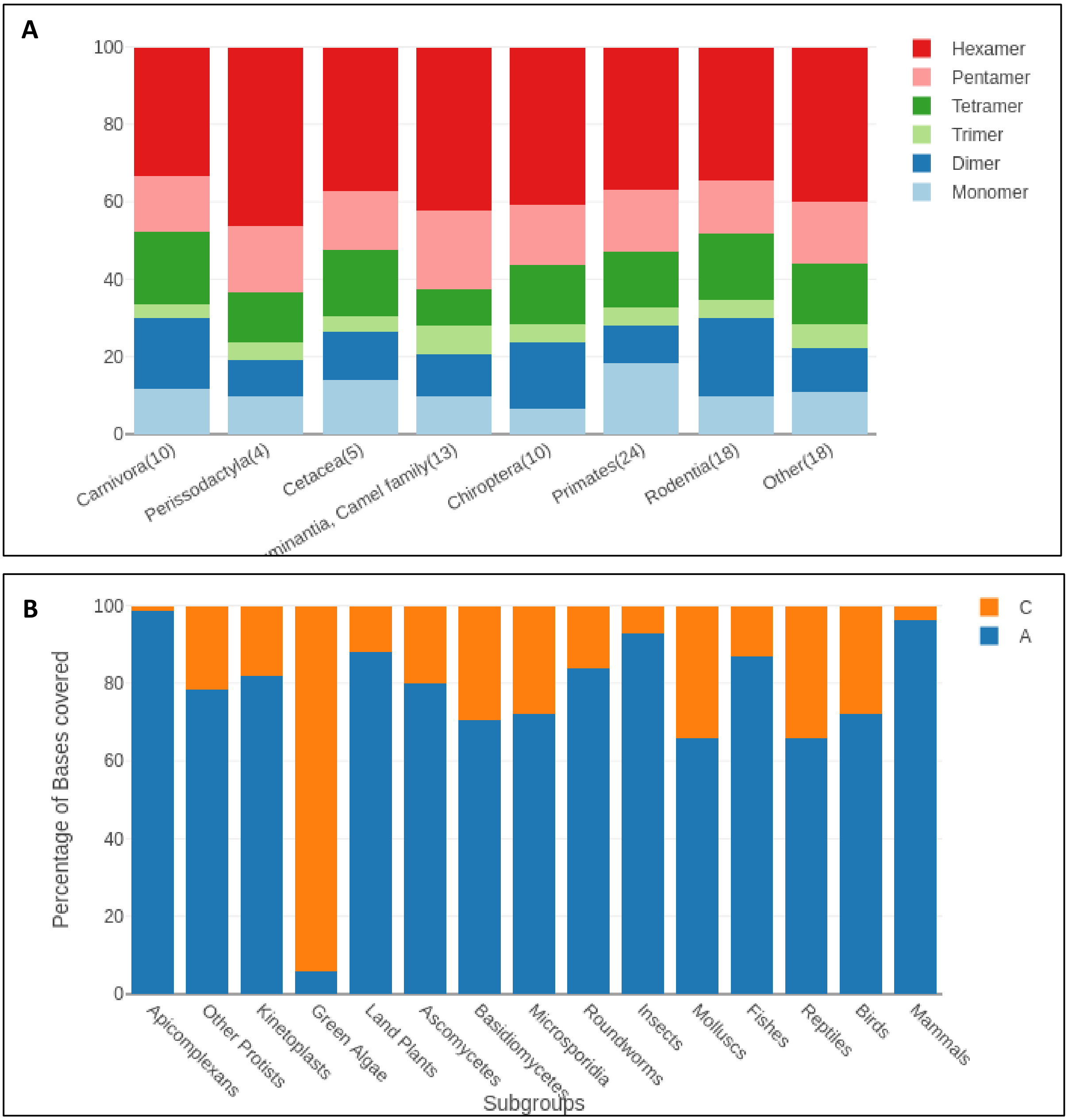

Figure S7: Composition of SSRs by motif size

For each subgroup/division, the number of bases covered by each SSR motif is calculated and divided by the total number of bases covered by all SSRs in that subgroup/division to get the percentage of each motif. **A)** SSR motif proportions in subdivisions of mammals. Rodents show the highest proportion (17%) of monomers in mammals (11.8%). **B)** PolyA versus polyC enrichment among monomers across subgroups. All subgroups are generally enriched in polyA repeats compared to polyC, except in green algae, where an inverse trend is seen

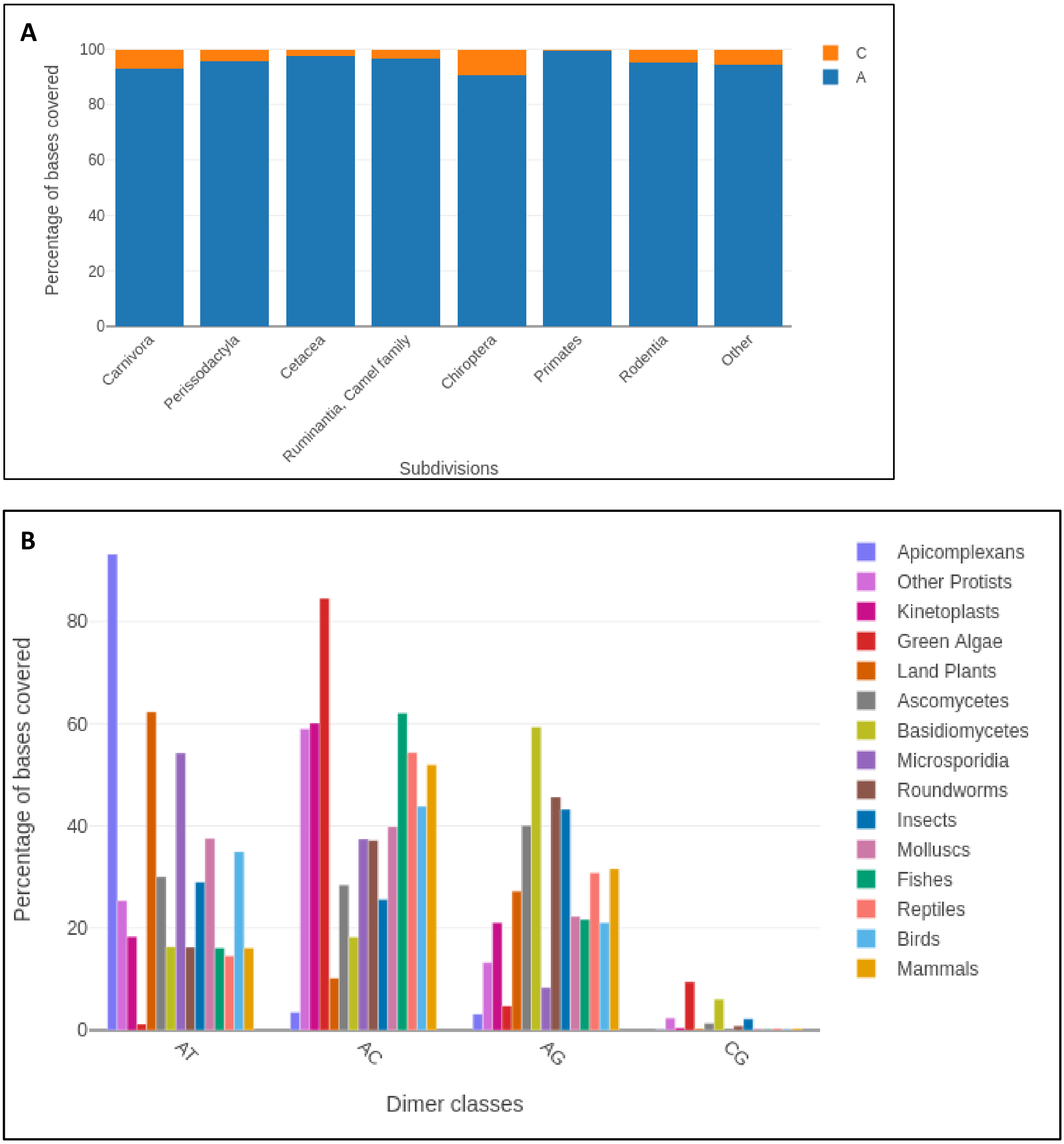

Figure S8: Composition of monomers and dimers by subgroup

For each subdivision, the number of bases covered by each SSR motif is calculated and divided by the total number of bases covered by all SSRs in that subgroup to get the percentage of each k-mer motif (Y-axis) in the subdivision (X-axis). **A)** PolyA versus polyC enrichment among monomers in subdivision of mammals. All mammals have high polyA content but in primates especially C monomers are almost absent. **B)** Relative proportions of dimers across subgroups**.** CG is generally absent in all species studied. No drastic differences are seen with respect to the distribution of dimers in most cases. Carnivores have a higher percentage of AG repeats compared to other groups, whereas birds have a marginally higher percentage of AT repeats.

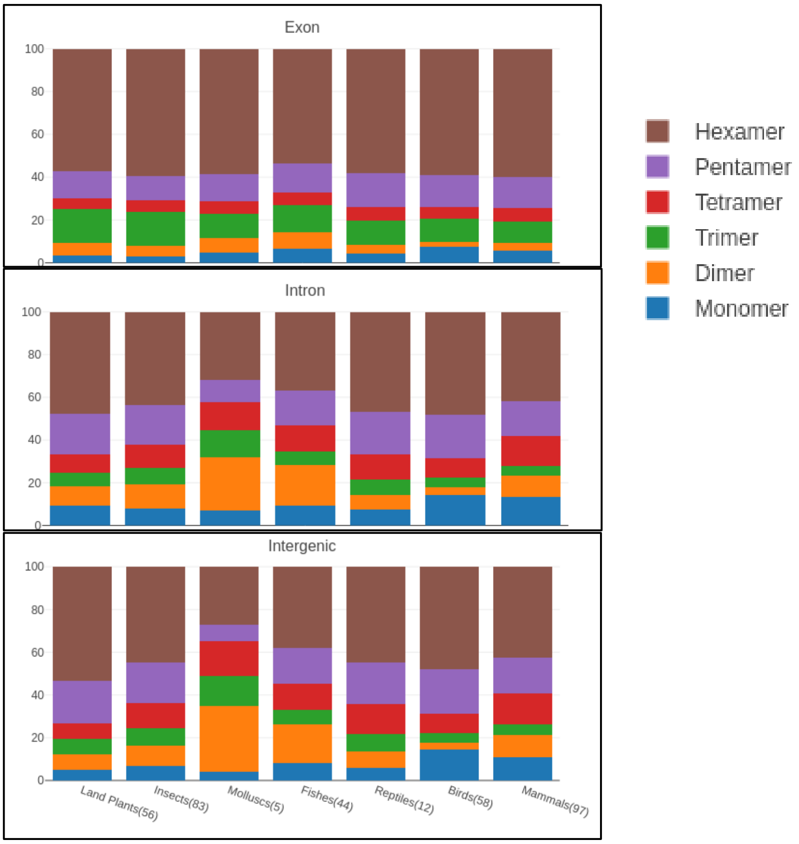

Figure S9: Motif distribution of SSRs among various genomic features. The fraction of each SSR motif is calculated as the number of SSRs of a given k-mer (motif) size overlapping various genomic features by the total number of SSRs overlapping the respective genomic features. This value is multiplied by 100 to derive percentages (the totals for all k-mer sizes add up to 100). The percentage of trimer and hexamer repeats is higher in exonic SSRs at the expense of tetramers and dimers. Notable differences include increased representation of dimers in intronic and intergenic regions of molluscs and fishes.
