# Supplementary material for: Patterns of microsatellite distribution across eukaryotic genomes
Source: BMC Genomics. 2019 Feb 22;20:153. doi: 10.1186/s12864-019-5516-5 (PMC6387519; doi:10.1186/s12864-019-5516-5)
Supplement: Supplementary file 4 — Table S3. Table of length preferences (DOCX 13 kb) [file 12864_2019_5516_MOESM4_ESM.docx]

**Supplementary table S3: Length preference ranges shown by repeats in subgroups**

| **Group** | **Subgroup** | **Length preference peak start-end (bp)** |
| --- | --- | --- |
| Protists | Apicomplexans | 44 - 56 |
|  | Other Protists | 56 - 69 |
|  | Kinetoplasts | 35 - 46 |
| Plants | Green Algae | 48 - 64 |
|  | Land Plants | 46 -58 |
| Fungi | Ascomycetes | 21 - 27 |
|  | Basidiomycetes | 16 - 21 |
|  | Microsporidia | NA |
| Invertebrates | Roundworms | 57 - 70 |
|  | Insects | 44 - 56 |
|  | Molluscs | 48 - 65 |
| Vertebrates | Fishes | 52 - 73 |
|  | Reptiles | 40 - 70 |
|  | Birds | 40 - 66 |
|  | Mammals | 36 - 60 |

Every instance of a repeat class showing preference for longer lengths in an organism was recorded (see Methods), and all organisms that belonged to the same subgroup were combined. The peak range of the subgroup was calculated by deriving the 5^th^ percentile of peak starts and 95^th^ percentile of peak ends within the subgroup to avoid outliers. NA indicates that not even a single instance of length preference was recorded for that subgroup.
